# Optimal timing of surgery after conversion therapy for initially unresectable hepatocellular carcinoma: a multicenter study integrating major pathological response and treatment duration

**DOI:** 10.3389/fimmu.2026.1911781

**Published:** 2026-09-21

**Authors:** Siming Li, Minjun Li, Fan Zhu, Yue Qin, Weiling Tang, JiaWen Yao, Jiahao Chen, Liang Liu, Zhiyin Liang, Huaning Wei, Yuxia He, Guang Wang, Jie Zhang, Bangde Xiang

**Affiliations:** 1 Department of Hepatobiliary Surgery, Guangxi Liver Cancer Diagnosis and Treatment Engineering and Technology Research Center, Guangxi Medical University Cancer Hospital, Nanning, China; 2 Department of Hepatobiliary and Pancreatic Surgery, The First Affiliated Hospital of Guilin Medical University, Guilin, Guangxi, China; 3 Department of Emergency Medicine, The First Affiliated Hospital of Guilin Medical University, Guilin, Guangxi, China; 4 Department of Hepatobiliary and Pancreatic Surgery, Wuming Hospital Affiliated to Guangxi Medical University, Nanning, Guangxi, China; 5 Department of Hepatobiliary and Pancreatic Surgery, Yulin Red Cross Hospital, Yulin, Guangxi, China; 6 Department of Hepatobiliary and Pancreatic Surgery, Yulin First People’s Hospital, Yulin, Guangxi Zhuang Autonomous Region, China; 7 Department of Hepatobiliary Surgery, The First Affiliated Hospital of Guangxi University of Chinese Medicine, Nanning, Guangxi, China; 8 Department of Hepatobiliary Surgery, The Second Nanning People’s Hospital, Nanning, China; 9 Department of Hepatobiliary Surgery, The First Hospital of China Medical University, Shenyang, Liaoning, China

**Keywords:** conversion therapy, hepatectomy, hepatocellular carcinoma, major pathological response, residual viable tumor, surgical timing

## Abstract

**Objective:**

The optimal timing of surgery after conversion therapy for initially unresectable hepatocellular carcinoma (uHCC) remains unclear. This study aimed to evaluate whether surgical timing can be optimized by integrating major pathological response (MPR), defined as residual viable tumor (RVT) ≤15%, with preoperative treatment duration.

**Methods:**

This multicenter retrospective study enrolled patients with initially uHCC who underwent curative-intent hepatectomy after comprehensive conversion therapy at seven tertiary hospitals between January 2020 and December 2025. The prognostic cutoff for MPR was determined using restricted cubic spline (RCS) models. Patients were categorized into three groups according to the interval from initiation of conversion therapy to surgery: <12 weeks, 12–24 weeks, and >24 weeks. MPR rates, survival outcomes, and perioperative safety were compared among the three groups.

**Results:**

A total of 257 patients were included. RCS analysis showed that a higher RVT proportion was associated with increased risks of poor overall survival (OS) and recurrence-free survival (RFS), with the most pronounced change in risk observed at an RVT threshold of 15%. Therefore, RVT ≤15% was defined as MPR in this study. The overall MPR rate was 63.4% (163/257). MPR was an independent protective factor for both OS and RFS. The MPR rate increased progressively with longer treatment duration (41.3% in the <12-week group, 66.2% in the 12–24-week group, and 73.0% in the >24-week group). OS differed significantly among the three duration groups, whereas the difference in RFS was not statistically significant. The incidences of severe postoperative complications and high-grade adverse events were low across all groups and did not increase markedly with prolonged therapy.

**Conclusions:**

RVT ≤15% may serve as a prognostically meaningful criterion for MPR after conversion therapy for uHCC. To balance the degree of pathological response, long-term survival benefit, and perioperative safety, a treatment-to-surgery interval of 12 to 24 weeks may be optimal.

## Introduction

Hepatocellular carcinoma (HCC) remains one of the most common malignancies worldwide and a leading cause of cancer-related mortality (1). Because of its insidious onset and lack of specific early symptoms, a substantial proportion of patients are diagnosed at an intermediate or advanced stage, when upfront curative resection is no longer feasible (2). In recent years, however, advances in locoregional therapy, molecular targeted therapy, and immune checkpoint inhibitors have substantially reshaped the therapeutic landscape of HCC (3). Multimodal conversion regimens, particularly those integrating transarterial chemoembolization (TACE), hepatic arterial infusion chemotherapy (HAIC), molecular targeted agents, and immunotherapy, have shown the potential to reduce tumor burden, induce downstaging, control vascular invasion, and ultimately render selected patients with initially unresectable HCC eligible for curative-intent resection (4–6). Accumulating evidence suggests that patients who successfully undergo hepatectomy after conversion therapy may achieve superior long-term survival compared with those who do not undergo surgery (7).

With the increasing success of conversion therapy, a clinically important yet unresolved question has emerged: once patients meet radiological or surgical criteria for resectability, should surgery be performed immediately, or should conversion therapy be continued to achieve a deeper antitumor response? At present, surgical decision-making after conversion therapy is mainly guided by radiological response, technical resectability, liver functional reserve, future liver remnant volume, and multidisciplinary team assessment (8). Although these parameters are essential for determining whether resection is feasible, they provide limited evidence regarding the optimal timing of surgery. Premature surgery may result in insufficient pathological response and a high proportion of residual viable tumor (RVT), thereby increasing the risk of postoperative recurrence (9). Conversely, excessive prolongation of conversion therapy may further deepen tumor response, but may also be accompanied by impaired liver function, cumulative treatment-related toxicity, increased operative difficulty, or even loss of the surgical window due to disease progression (10, 11). Therefore, balancing the depth of pathological response against the risks of treatment delay represents a key challenge in surgical decision-making after conversion therapy.

Pathological response provides a valuable perspective for addressing this issue. Unlike radiological response, postoperative pathological evaluation directly reflects the proportion of RVT, treatment-induced necrosis, fibrosis, and tumor-bed remodeling (9). With the growing use of neoadjuvant and conversion therapy in HCC, pathological complete response (pCR) and major pathological response (MPR) have increasingly been used to quantify the depth of antitumor response and to evaluate pathological efficacy and prognostic risk (12). Previous studies have shown that among patients with initially unresectable HCC who receive combination conversion therapy followed by resection, those achieving pCR or MPR generally experience improved overall survival (OS) and recurrence-free survival (RFS) (13). These findings suggest that the depth of pathological response may serve as an important link between preoperative treatment efficacy and long-term postoperative outcomes.

Nevertheless, the definition and clinical application of MPR in HCC remain insufficiently standardized. Some studies define MPR as RVT ≤10% or pathological response ≥90%, whereas others adopt thresholds such as tumor necrosis ≥70% (14, 15). Importantly, MPR can identify patients who have not achieved pCR but may still derive substantial survival benefit, thereby capturing a broader spectrum of prognostic heterogeneity than pCR alone (16). Current expert consensus emphasizes that surgical timing after conversion therapy should integrate tumor response, liver function, future liver remnant volume, general condition, and treatment safety (17). However, quantitative evidence based on the combined assessment of pathological response depth and treatment duration remains limited.

Therefore, integrating MPR with the interval from initiation of conversion therapy to surgery may provide a more comprehensive understanding of the true benefit of conversion therapy than either radiological response or treatment duration alone. On the one hand, treatment duration reflects cumulative exposure to conversion therapy and the potential process of response deepening (18). On the other hand, MPR reflects the extent of tumor clearance at the pathological level (19). Combining these two dimensions may help answer several clinically relevant questions: Does a longer treatment duration truly translate into a deeper pathological response? Does an increased probability of MPR necessarily lead to improved survival? Is there an optimal treatment-to-surgery window that maximizes the likelihood of MPR while avoiding unnecessary treatment delay, postoperative recurrence, mortality, or increased perioperative risk? Addressing these questions is essential for optimizing surgical decision-making after conversion therapy and for avoiding both premature resection and ineffective prolongation of treatment.

In the present study, we enrolled patients with initially unresectable HCC who successfully underwent curative-intent resection after conversion therapy. We systematically evaluated the relationships among RVT proportion, MPR, duration of conversion therapy, postoperative recurrence, and survival outcomes. We further explored MPR rates, recurrence risk, and OS across different treatment-duration intervals, aiming to provide evidence for optimizing surgical timing based on both pathological response depth and treatment duration. This study seeks to shift surgical decision-making after conversion therapy from simply determining whether resection is feasible to identifying when resection is most appropriate, thereby supporting individualized surgical planning for patients with initially unresectable HCC.

## Materials and methods

### Patients

This multicenter retrospective cohort study consecutively enrolled patients with initially uHCC who received conversion therapy and successfully underwent curative-intent hepatectomy at seven tertiary hospitals between January 2020 and December 2025. At initial diagnosis, all patients were evaluated by a multidisciplinary team (MDT) and were considered unresectable or unsuitable for upfront curative resection. After comprehensive conversion therapy based on locoregional therapy, molecular targeted therapy, and immune checkpoint inhibitors, patients were reassessed; those who met surgical criteria subsequently underwent curative-intent hepatectomy. Because this study was restricted to patients who were successfully converted and underwent surgical resection, the conclusions should be interpreted primarily within this population. This study was conducted in accordance with the ethical principles of the Declaration of Helsinki and was approved by the ethics committees of the participating centers.

The inclusion criteria were as follows: 1) age ≥18 years; 2) diagnosis of hepatocellular carcinoma based on clinical, radiological, or pathological criteria (20); 3) initially unresectable disease as assessed by a multidisciplinary team before treatment; 4) receipt of preoperative triple conversion therapy consisting of locoregional therapy, molecular targeted therapy, and immune checkpoint inhibitors; 5) successful conversion to surgical eligibility followed by curative-intent hepatectomy; and 6) availability of complete clinical, radiological, treatment, surgical, pathological, and follow-up data.

The exclusion criteria were as follows: 1) absence of preoperative triple conversion therapy; 2) receipt of single-modality therapy or non-triple combination therapy before surgery; 3) initially resectable disease; 4) receipt of palliative surgery, exploratory surgery, ablation; 5) coexistence of other malignancies; 6) death from any cause within 90 days after surgery;7)missing key information regarding preoperative treatment, treatment duration, surgical date, pathological assessment, or survival follow-up (**Figure 1**).

**Figure 1:**
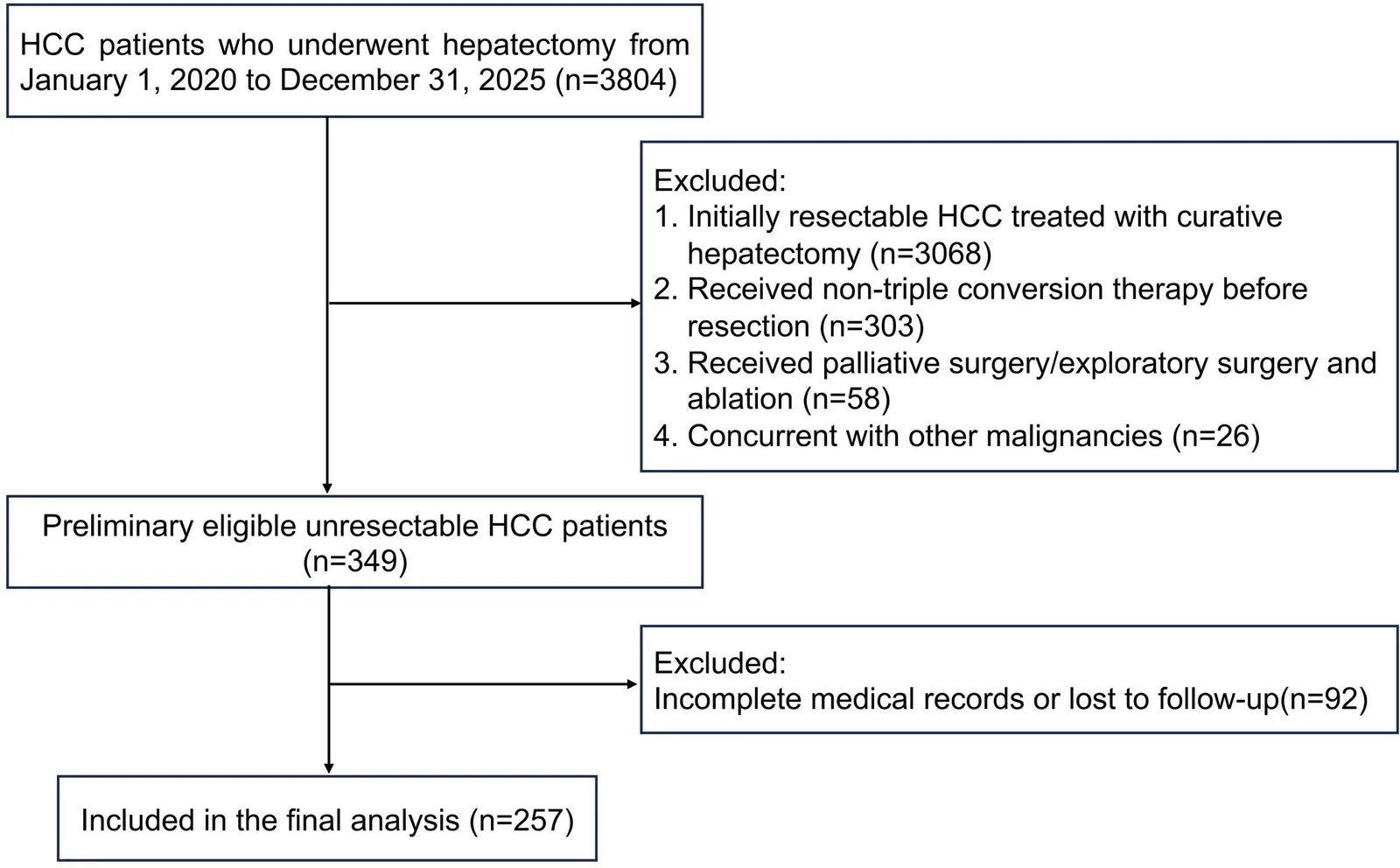
Patient selection flowchart.

### Definition of initially uHCC

Initially unresectable HCC was defined as HCC considered unsuitable for upfront curative hepatectomy before the first antitumor treatment, based on MDT assessment of tumor burden, tumor distribution, vascular invasion, anatomical location, hepatic functional reserve, or insufficient future liver remnant, but with potential eligibility for subsequent curative-intent resection after conversion therapy. Unresectable factors included, but were not limited to, large tumor burden or multifocal/bilobar disease precluding R0 resection at the initial stage; invasion of major vessels, such as the portal vein, hepatic vein, or inferior vena cava; tumors adjacent to critical vascular or biliary structures that increased the risk of direct resection; inadequate hepatic functional reserve or insufficient future liver remnant; and BCLC stage B or C or CNLC stage IIb–IIIa disease requiring conversion therapy as determined by MDT assessment. Initial resectability was jointly determined by specialists in hepatobiliary surgery, medical oncology, interventional radiology, radiology, radiation oncology, and pathology.

### Conversion therapy regimens

TACE was performed via right femoral artery puncture and microcatheter placement. Under imaging guidance, the microcatheter was superselectively advanced into the tumor-feeding arteries, followed by chemoembolization. For patients with multiple intrahepatic tumors, superselective embolization was performed for visible tumor-feeding arteries identified by selective angiography; lesions not visible on angiography were not routinely embolized. Oxaliplatin was thoroughly mixed with iodized oil and injected through the microcatheter into the selected tumor-feeding arteries, followed by embolization with gelatin sponge particles. After treatment, alpha-fetoprotein (AFP), protein induced by vitamin K absence or antagonist-II (PIVKA-II), and contrast-enhanced computed tomography (CT) or magnetic resonance imaging (MRI) were assessed every 4 weeks to evaluate tumor response and tumor vascularity. If imaging suggested persistent arterial blood supply and the patient could tolerate treatment, TACE was repeated every 4–6 weeks as clinically indicated.

HAIC was performed via right femoral artery puncture and microcatheter placement. Under digital subtraction angiography (DSA) guidance, the microcatheter was advanced into the hepatic arterial trunk or tumor-feeding arterial branches. For patients with multiple intrahepatic tumors or extensive tumor involvement, the infusion territory was determined according to the hepatic arterial trunk and major tumor-feeding branches identified by selective angiography, with the aim of covering visible tumor regions and potential microscopic lesions. Fluoropyrimidines, such as floxuridine or 5-fluorouracil, combined with platinum agents, such as oxaliplatin or cisplatin, were continuously infused into the target artery through the microcatheter to achieve high local drug concentrations within the tumor. After treatment, AFP, liver function, and contrast-enhanced CT or MRI were assessed every 3–4 weeks to evaluate tumor size, enhancement, and changes in hepatic arterial supply. If imaging showed persistent active tumor vascularity and the patient tolerated treatment, HAIC was repeated every 4–6 weeks as clinically indicated until the best response was achieved or intolerable adverse events occurred. All patients with active hepatitis B virus infection received standard oral antiviral therapy.

Systemic therapy included molecular targeted agents, antiangiogenic agents, and immune checkpoint inhibitors. The specific agents and treatment combinations were selected by the multidisciplinary team in accordance with contemporaneous clinical guidelines and real-world clinical practice. Treatment selection was individualized based on liver function, performance status, tumor burden, vascular invasion, comorbidities, drug contraindications, anticipated adverse events, previous treatment, drug availability, financial circumstances, patient preferences, and physician experience. Lenvatinib was administered according to body weight: 12 mg once daily for patients weighing ≥60 kg and 8 mg once daily for those weighing <60 kg. Donafenib was administered at 0.2 g twice daily. Apatinib was administered at 750 mg once daily, 30 minutes after meals, and could be reduced to 500 mg/day in patients with poor tolerance. Bevacizumab was administered at 15 mg/kg every 3 weeks. Sintilimab and tislelizumab were each administered at 200 mg every 3 weeks. Tyrosine kinase inhibitors and programmed cell death protein 1 (PD-1) inhibitors were generally initiated within 3–14 days after the first interventional treatment and withheld for 3 days before and after subsequent interventional procedures. Treatment-related adverse events were graded according to the Common Terminology Criteria for Adverse Events (CTCAE), version 5.0, and managed with symptomatic supportive care, dose adjustment, or treatment interruption according to standard clinical practice.During conversion therapy, patients regularly underwent contrast-enhanced CT or MRI, liver function tests, coagulation tests, complete blood counts, AFP measurements, and other relevant examinations. Radiological response was mainly evaluated according to RECIST version 1.1 and/or modified RECIST criteria.

Surgery was considered after MDT reassessment when the following criteria were met (1): marked tumor shrinkage or necrosis resulting in technical resectability (2); regression or disappearance of vascular invasion, or vascular invasion amenable to combined resection and reconstruction (3); absence of uncontrolled extrahepatic metastasis or systemic progression (4); Child-Pugh class A liver function or liver function deemed sufficient to tolerate surgery (5); adequate future liver remnant volume (6); performance status compatible with curative-intent surgery; and (7) anticipated achievement of R0 resection or curative-intent resection.

Surgical procedures included anatomical or non-anatomical hepatectomy, with the specific approach determined by the surgeon according to tumor location, tumor extent, vascular relationships, and hepatic functional reserve. Portal vein tumor thrombus extraction, vascular resection and reconstruction, biliary reconstruction, or adjacent organ/tissue resection was performed when necessary. Operative time, intraoperative blood loss, transfusion, extent of resection, resection margin status, postoperative complications, and length of hospital stay were recorded.

### Multicenter pathological assessment

All resected specimens were processed according to a standardized multicenter pathological assessment protocol. Specimens were fixed in 10% neutral buffered formalin, grossly examined, and sampled by the pathology department at each participating center, with centralized pathological review and quality control performed by the leading center. Tumors ≤3 cm were entirely sampled, whereas tumors >3 cm were serially sectioned at 5–10-mm intervals. Representative tissue samples were obtained from the tumor bed, areas suspected of containing residual viable tumor (RVT), necrotic regions, tumor margins, sites of vascular invasion, the closest resection margin, and non-tumorous liver tissue. RVT was defined as the proportion of morphologically identifiable viable tumor within the entire tumor-bed area. The tumor-bed area comprised residual viable tumor, necrosis, hemorrhage, fibrosis, inflammatory infiltration, and post-treatment scar-like changes. For multifocal tumors, the overall RVT was calculated using an area-weighted method. Specifically, the tumor-bed area and residual viable tumor area of each lesion were independently delineated and quantified on hematoxylin and eosin-stained sections. The residual viable tumor areas of all lesions were then summed and divided by the sum of the corresponding tumor-bed areas. Overall RVT was calculated as follows:

Overall RVT (%) = (Σ residual viable tumor area of all lesions/Σ tumor-bed area of all lesions) × 100%.

Accordingly, the contribution of each lesion to the overall RVT estimate was proportional to its tumor-bed area, with larger lesions contributing greater weight than smaller lesions. Pathological complete response (pCR) was defined as the absence of viable tumor cells in all resected lesions and corresponding tumor beds.All cases were independently reviewed by two trained liver pathologists who were blinded to the patients’ survival outcomes. Discrepant cases, defined as an absolute difference of >10 percentage points in RVT estimation or disagreement regarding pCR, MPR, microvascular invasion, or margin status, were adjudicated by a third senior pathologist. The final pathological variables were determined based on the centralized review and adjudication results.

### Follow-up and study endpoints

Follow-up assessments included abdominal contrast-enhanced CT or MRI, AFP measurement, liver function assessment, and other imaging or laboratory examinations when necessary. During conversion therapy, efficacy and safety were evaluated every 4–8 weeks. For patients who developed progressive disease (PD), intolerable toxicity, or withdrawal of consent, subsequent treatment strategies were determined after thorough discussion between the MDT and the patient. Patients without disease progression who tolerated therapy continued conversion therapy until the best response, symptomatic progression, intolerable toxicity, or withdrawal of consent. Postoperative follow-up and subsequent treatment strategies were also determined after MDT discussion.

The primary endpoint was OS, defined as the time from initiation of the first conversion treatment to death from any cause. Patients who were alive at the last follow-up were censored at the date of last valid follow-up. The secondary endpoint was RFS, defined as the time from curative-intent hepatectomy to the first radiologically or pathologically confirmed tumor recurrence or death from any cause. Patients without recurrence or death during follow-up were censored at the date of last valid follow-up. The last follow-up date for all patients was April 1, 2026.

### Statistical analysis

Continuous variables are presented as mean ± standard deviation and were compared between groups using Student’s t test. Categorical variables are presented as number and percentage [n (%)] and were compared using the chi-square test or Fisher’s exact test, as appropriate. Patients were classified into MPR and non-MPR groups according to postoperative RVT proportion, and baseline clinicopathological characteristics were compared between the two groups.

RCS models were used to evaluate the continuous association between RVT proportion and the risks of adverse OS and RFS outcomes. The MPR cutoff was determined according to the risk-change pattern and clinical interpretability. Preoperative conversion therapy duration was defined as the interval from initiation of the first conversion treatment to hepatectomy and was categorized as <12 weeks, 12–24 weeks, or >24 weeks. OS and RFS curves were generated using the Kaplan-Meier method, and survival differences between groups were compared using the log-rank test. To further evaluate the relationships among preoperative conversion therapy duration, pathological response, and prognosis, MPR rate, OS, RFS, perioperative complications, and treatment-related AEs were compared across the three treatment-duration groups.

Univariable Cox proportional hazards regression analysis was used to screen potential prognostic factors associated with OS and RFS. Variables with P < 0.05 in univariable analysis were included in multivariable Cox proportional hazards regression models to identify independent prognostic factors. Risks are presented as hazard ratios (HRs) with corresponding 95% confidence intervals (CIs). All statistical tests were two-sided, and P < 0.05 was considered statistically significant. All statistical analyses were performed using R version 4.5.2.

## Results

### Association between RVT proportion and prognosis and determination of the MPR cutoff

To evaluate the relationship between postoperative RVT proportion and long-term outcomes, RCS models were used to analyze the continuous associations between RVT proportion and the risks of poor OS and RFS. The results showed that the risks of adverse OS and RFS outcomes increased with increasing RVT proportion. The risk curves showed an evident change at an RVT proportion of approximately 15%, beyond which the increase in risk gradually plateaued. Therefore, an RVT proportion of 15% was selected as the pathological response stratification cutoff in this study, with RVT ≤15% defined as MPR and RVT >15% defined as non-MPR (**Figure 2**).

**Figure 2:**
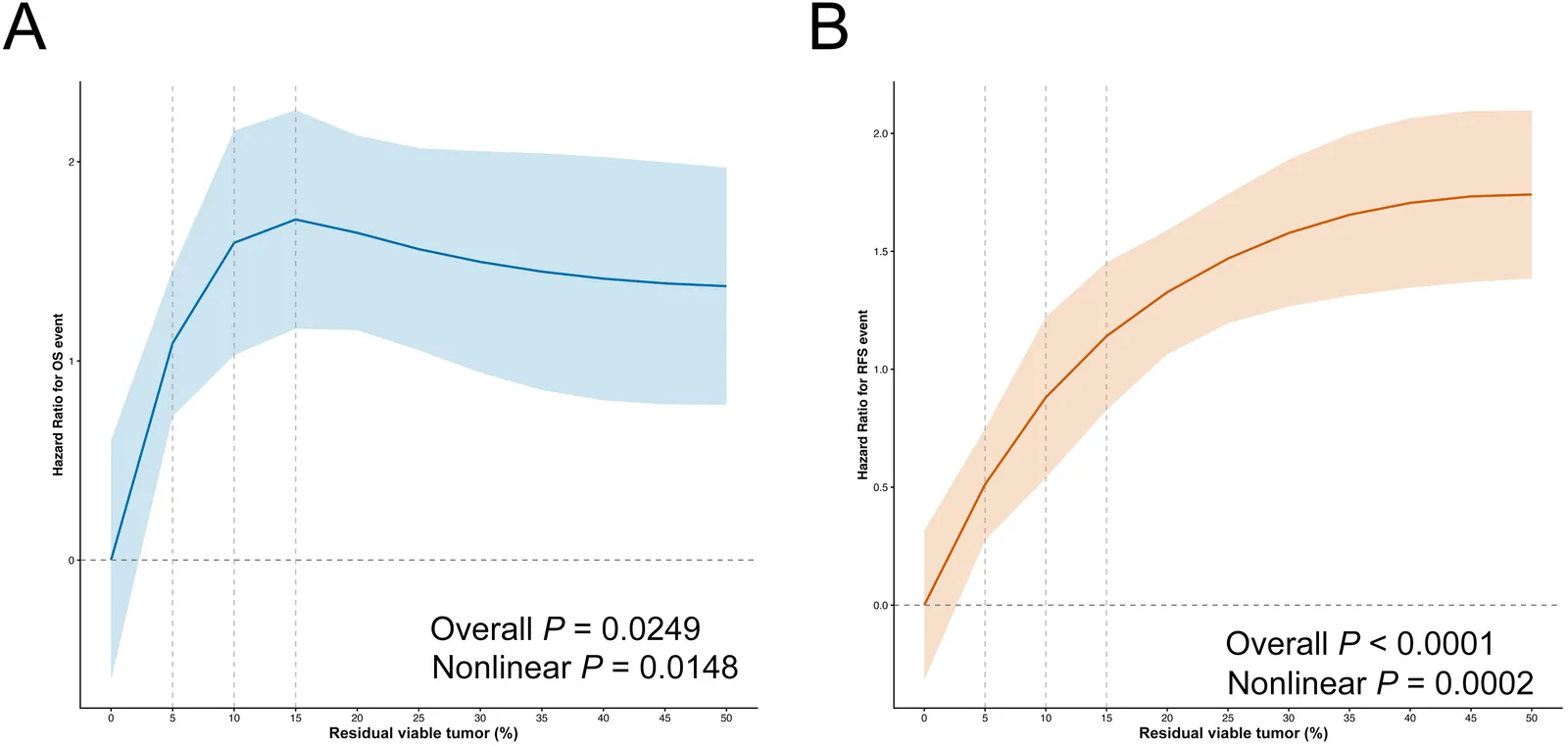
Restricted cubic spline analysis of optimal cutoff value of residual viable tumor for predicting overall survival **(A)** and recurrence-free survival **(B)** risk.

### Baseline characteristics

A total of 257 patients with initially uHCC who underwent surgical resection after conversion therapy were included, comprising 163 patients (63.4%) in the MPR group and 94 patients (36.6%) in the non-MPR group. In the overall cohort, 227 patients (88.3%) were male and 30 (11.7%) were female; 210 patients (81.7%) were aged ≤60 years and 47 (18.3%) were aged >60 years. Hepatitis B virus infection was present in 221 patients (86.0%), and cirrhosis was present in 216 patients (84.0%). Child-Pugh class A liver function was observed in 248 patients (96.5%), whereas 9 patients (3.5%) had Child-Pugh class B liver function. BCLC stages A, B, and C were observed in 74 (28.8%), 71 (27.6%), and 112 patients (43.6%), respectively. Tumor diameter ≥5 cm was observed in 234 patients (91.1%).

There were no statistically significant differences between the MPR and non-MPR groups in sex, age, alcohol consumption history, body mass index, Child-Pugh grade, diabetes mellitus, hepatitis B virus infection, cirrhosis, alanine aminotransferase, aspartate aminotransferase, or postoperative adjuvant therapy. Compared with the non-MPR group, the MPR group had a lower proportion of patients with preoperative AFP ≥400 ng/mL (9.2% vs. 20.2%, P = 0.02). The distribution of BCLC stage differed significantly between the two groups (P = 0.002), with a higher proportion of BCLC stage C patients in the MPR group (51.5% vs. 29.8%). In addition, the proportion of patients with tumor diameter ≥5 cm was higher in the MPR group than in the non-MPR group (94.5% vs. 85.1%, P = 0.02). Regarding pathological characteristics, the distribution of MVI grade differed significantly between the two groups (P<0.001) (**Table 1**).

**Table 1:** Baseline characteristics of uHCC patients receiving triple conversion therapy.

Variables	MPR group (n=163)	Non-MPR group (n=94)	*P* value
Age, year			0.91
≤60	134 (82.2)	76 (80.9)	
>60	29 (17.8)	18 (19.1)	
Sex			0.53
Male	146 (89.6)	81 (86.2)	
Female	17 (10.4)	13 (13.8)	
BMI, kg/m^2^			0.31
<24	103 (63.2)	66 (70.2)	
≥24	60 (36.8)	28 (29.8)	
Diabetes			1.00
Present	10 (6.1)	5 (5.3)	
Absent	153 (93.9)	89 (94.7)	
Alcohol intake			0.43
Present	60 (36.8)	40 (42.6)	
Absent	103 (63.2)	54 (57.4)	
HBV infection			0.53
Present	138 (84.7)	83 (88.3)	
Absent	25 (15.3)	11 (11.7)	
Liver cirrhosis			0.86
Present	136 (83.4)	80 (85.1)	
Absent	27 (16.6)	14 (14.9)	
Preoperative AFP, ng/mL			0.02
≥400	15 (9.2)	19 (20.2)	
<400	148 (90.8)	75 (79.8)	
AFP, ng/mL			0.065
≥400	85 (52.1)	37 (39.4)	
<400	78 (47.9)	57 (60.6)	
Microvascular invasion			<0.001
M0	150 (92.0)	67 (71.3)	
M1	9 (5.5)	14 (14.9)	
M2	4 (2.5)	13 (13.8)	
Child-Pugh grade			1.00
A	157 (96.3)	91 (96.8)	
B	6 (3.7)	3 (3.2)	
Tumor size, cm			0.021
≥5	154 (94.5)	80 (85.1)	
<5	9 (5.5)	14 (14.9)	
ALT, U/L			0.32
≥40	75 (46.0)	50 (53.2)	
<40	88 (54.0)	44 (46.8)	
AST, U/L			0.62
≥40	110 (67.5)	67 (71.3)	
<40	53 (32.5)	27 (28.7)	
Postoperative adjuvant therapy			0.86
Present	80 (49.1)	48 (51.1)	
Absent	83 (50.9)	46 (48.9)	
BCLC stage			0.002
A	43 (26.4)	31 (33.0)	
B	36 (22.1)	35 (37.2)	
C	84 (51.5)	28 (29.8)	

AFP, alpha-fetoprotein; ALT, Alanine aminotransferase; AST, aspartate transaminase; BCLC, Barcelona Liver Cancer staging; BMI, body mass index.

### MPR according to conversion therapy regimen

The MPR rates according to the different conversion therapy regimens are summarized in **Table 2**. Among the 257 patients, 149 received TACE combined with an ICI and a TKI, of whom 93 (62.4%) achieved MPR. Seventeen patients received TACE combined with an ICI and an anti-VEGF agent, with an MPR rate of 64.7% (11/17). Among patients receiving HAIC-based conversion therapy, MPR was achieved in 51 of 79 patients (64.6%) treated with an ICI plus a TKI and in 8 of 12 patients (66.7%) treated with an ICI plus an anti-VEGF agent. Overall, 163 patients (63.4%) achieved MPR. The MPR rates were numerically similar across the four treatment combinations, ranging from 62.4% to 66.7%, suggesting that a favorable pathological response was observed across both TACE- and HAIC-based triple conversion regimens.

**Table 2:** Major pathological response rates according to conversion therapy regimen.

Locoregional therapy	Immune checkpoint inhibitor	Targeted/antiangiogenic agent	Total, n	MPR, n (%)
TACE	ICI	TKI	149	93 (62.4)
TACE	ICI	Anti-VEGF	17	11 (64.7)
HAIC	ICI	TKI	79	51 (64.6)
HAIC	ICI	Anti-VEGF	12	8 (66.7)
**Overall**	**—**	**—**	**257**	**163 (63.4)**

Data are presented as n (%). 5-FU, 5-fluorouracil; HAIC, hepatic arterial infusion chemotherapy; ICI, immune checkpoint inhibitor; MPR, major pathological response; TACE, transarterial chemoembolization; TKI, tyrosine kinase inhibitor; VEGF, vascular endothelial growth factor.

Treatment details: TACE and HAIC regimens comprised oxaliplatin plus 5-FU. TKIs included lenvatinib mesylate, apatinib mesylate, and donafenib mesylate; the anti-VEGF agent was bevacizumab. ICIs included camrelizumab, sintilimab, and tislelizumab.

### Association of MPR with OS and RFS

Kaplan-Meier survival analysis showed that OS was significantly better in the MPR group than in the non-MPR group. Compared with patients with RVT >15%, those with RVT ≤15% had a significantly lower risk of death (HR = 0.25, 95% CI: 0.12–0.55, P < 0.0001). The MPR group also showed significantly better RFS. Patients with RVT ≤15% had a markedly lower risk of recurrence than those with RVT >15% (HR = 0.34, 95% CI: 0.22–0.54, P < 0.0001) (**Figure 3**).

**Figure 3:**
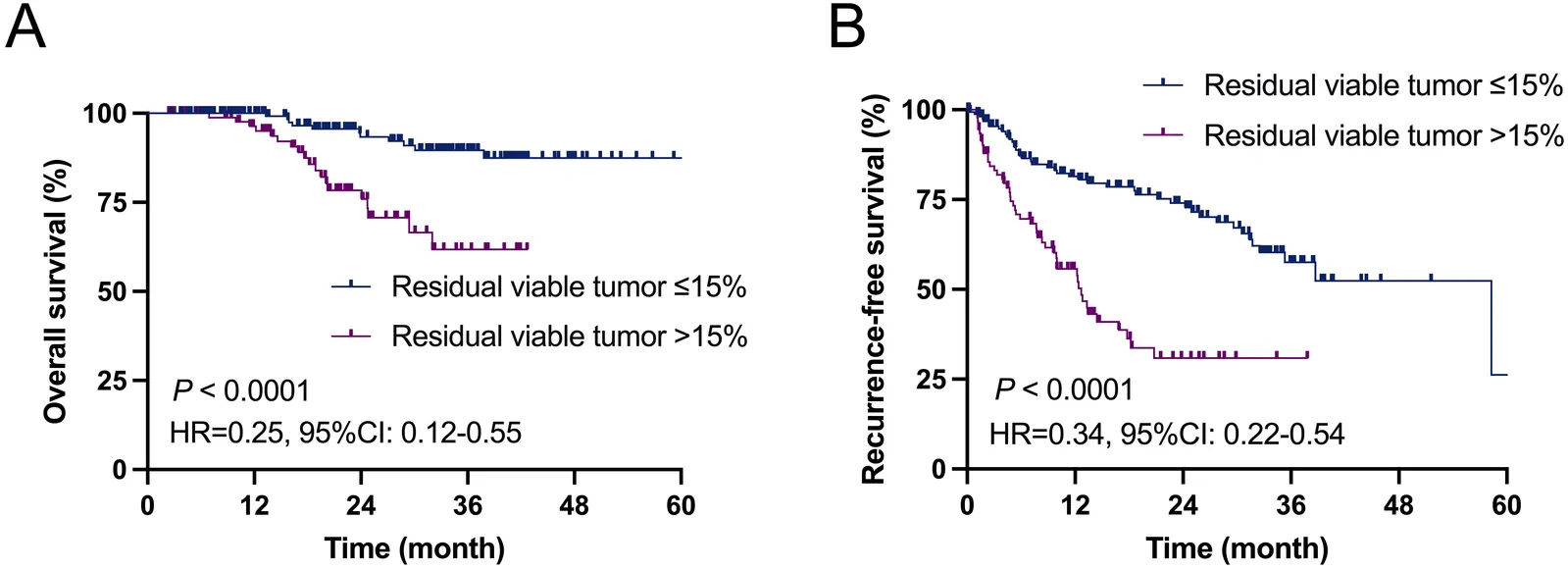
Kaplan-Meier analysis of overall survival **(A)** and recurrence-free survival **(B)** between MPR group and non-MPR group.

### Preoperative conversion therapy duration, MPR rate, and survival outcomes

Preoperative conversion therapy duration was defined as the interval from initiation of the first conversion treatment to hepatectomy. Patients were divided into three groups: <12 weeks, 12–24 weeks, and >24 weeks. The MPR rates were 41.3%, 66.2%, and 73.0%, respectively, showing a progressive increase with longer treatment duration. Kaplan-Meier analysis showed a significant difference in OS among the three treatment-duration groups (P = 0.04), with the 12–24-week group showing the most favorable survival trend. However, the difference in RFS among the three groups was not statistically significant (P = 0.19) (**Figure 4**).

**Figure 4:**
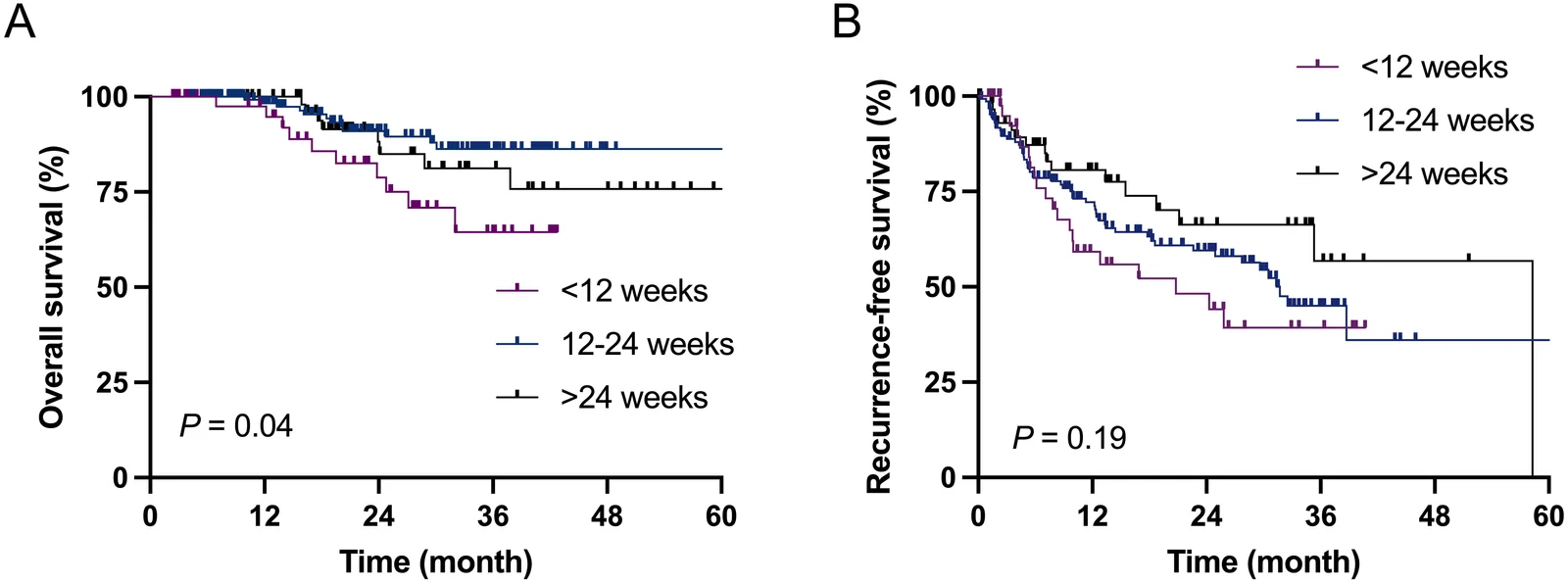
Kaplan-Meier analysis of overall survival **(A)** and recurrence-free survival **(B)** among patients treated with different preoperative conversion therapy duration.

### Risk factors associated with OS

In univariable Cox regression analysis for OS, age ≤60 years was associated with a lower risk of death (HR = 0.41, 95% CI: 0.19–0.88, P = 0.02); preoperative AFP ≥400 ng/mL was associated with a higher risk of death (HR = 3.29, 95% CI: 1.40–7.70, P = 0.006); MPR was significantly associated with a lower risk of death (HR = 0.24, 95% CI: 0.11–0.52, P < 0.001); and MVI-M2 was associated with a higher risk of death (HR = 3.23, 95% CI: 1.22–8.59, P = 0.01).

Multivariable Cox regression analysis showed that age ≤60 years remained a protective factor for OS (HR = 0.36, 95% CI: 0.16–0.81, P = 0.014), preoperative AFP ≥400 ng/mL was an independent risk factor for poor OS (HR = 3.13, 95% CI: 1.16–8.42, P = 0.02), and MPR was an independent protective factor for OS (HR = 0.26, 95% CI: 0.12–0.58, P = 0.001).

### Risk factors associated with RFS

In univariable Cox regression analysis for RFS, preoperative AFP ≥400 ng/mL was associated with a higher risk of recurrence (HR = 1.98, 95% CI: 1.13–3.46, P = 0.016); MPR was significantly associated with a lower risk of recurrence (HR = 0.31, 95% CI: 0.20–0.48, P < 0.001); and both MVI-M1 and MVI-M2 were associated with higher recurrence risks, with HRs of 2.63 (95% CI: 1.47–4.72, P = 0.001) and 2.90 (95% CI: 1.56–5.37, P < 0.001), respectively. In addition, BCLC stage B was associated with a higher risk of recurrence (HR = 2.15, 95% CI: 1.24–3.72, P = 0.006), and postoperative adjuvant therapy was also associated with worse RFS (HR = 1.65, 95% CI: 1.07–2.55, P = 0.02).

Multivariable Cox regression analysis showed that MPR remained an independent protective factor for RFS (HR = 0.32, 95% CI: 0.20–0.53, P < 0.001). MVI-M1 was an independent risk factor for worse RFS (HR = 2.14, 95% CI: 1.15–3.98, P = 0.016). Both BCLC stage B and BCLC stage C were independently associated with higher recurrence risk, with HRs of 2.77 (95% CI: 1.55–4.94, P = 0.001) and 2.11 (95% CI: 1.20–3.71, P = 0.01), respectively. Postoperative adjuvant therapy was also associated with RFS events (HR = 1.83, 95% CI: 1.16–2.90, P = 0.009). This finding indicates a statistical association between postoperative adjuvant therapy and RFS events, but it should not be interpreted as evidence that adjuvant therapy itself increases the risk of recurrence (**Table 3**).

**Table 3:** Risk factors of overall and recurrence-free survival for uHCC patients.

Variable	Overall survival				Recurrence-free survival			
	Univariate analysis		Multivariable analysis		Univariate analysis		Multivariable analysis	
	HR (95% CI)	*P* value	HR (95% CI)	*P* value	HR (95% CI)	*P* value	HR (95% CI)	*P* value
Age, year								
>60	1.00				1.00			
≤60	0.41 (0.19-0.88)	0.02	0.36 (0.16-0.81)	0.014	1.06 (0.62-1.82)	0.82		
Sex								
Female	1.00				1.00			
Male	3.73 (0.51-27.37)	0.19			0.66 (0.37-1.19)	0.16		
BMI, kg/m^2^								
<24	1.00				1.00			
≥24	0.84 (0.38-1.83)	0.66			1.00 (0.65-1.54)	0.99		
Diabetes								
Absent	1.00				1.00			
Present	1.22 (0.29-5.11)	0.79			1.45 (0.67-3.13)	0.34		
ALT, U/L								
<40	1.00				1.00			
≥40	0.75 (0.36-1.55)	0.43			1.08 (0.72-1.63)	0.71		
AST, U/L								
<40	1.00				1.00			
≥40	1.00 (0.46-2.18)	0.99			0.76 (0.50-1.17)	0.21		
Child-Pugh grade								
A	1.00				1.00			
B	3.84 (0.90-16.30)	0.06			0.44 (0.06-3.14)	0.41		
Tumor size, cm								
<5	1.00				1.00			
≥5	1.51 (0.36-6.36)	0.57			0.57 (0.32-1.03)	0.06		
Liver cirrhosis								
Absent	1.00				1.00			
Present	1.41 (0.43-4.66)	0.56			1.71 (0.83-3.53)	0.14		
AFP, ng/mL								
<400	1.00				1.00			
≥400	0.97 (0.47-1.98)	0.93			0.83 (0.55-1.25)	0.36		
Preoperative AFP, ng/mL								
<400	1.00				1.00			
≥400	3.29 (1.40-7.70)	0.006	3.13 (1.16-8.42)	0.02	1.98 (1.13-3.46)	0.016	1.71 (0.87-3.35)	0.11
HBV infection								
Absent	1.00				1.00			
Present	1.11 (0.15-8.20)	0.920			0.71 (0.35-1.45)	0.34		
MPR group								
non-MPR	1.00				1.00			
MPR	0.24 (0.11-0.52)	<0.001	0.26 (0.12-0.58)	0.001	0.31 (0.20-0.48)	<0.001	0.32 (0.20-0.53)	<0.001
Microvascular invasion								
M0	1.00				1.00			
M1	1.76 (0.60-5.18)	0.30	0.91 (0.30-2.78)	0.87	2.63 (1.47-4.72)	0.001	2.14 (1.15-3.98)	0.016
M2	3.23 (1.22-8.59)	0.01	1.29 (0.38-4.37)	0.67	2.90 (1.56-5.37)	<0.001	1.89 (0.88-4.08)	0.104
BCLC stage								
A	1.00				1.00			
B	2.29 (0.86-6.11)	0.09			2.15 (1.24-3.72)	0.006	2.77 (1.55-4.94)	0.001
C	1.60 (0.60-4.27)	0.34			1.42 (0.83-2.42)	0.20	2.11 (1.20-3.71)	0.010
Postoperative adjuvant therapy								
Absent	1.00				1.00			
Present	1.04 (0.50-2.17)	0.91			1.65 (1.07-2.55)	0.02	1.83 (1.16-2.90)	0.009

AFP, alpha-fetoprotein; ALT, Alanine aminotransferase; AST, aspartate transaminase; BCLC, Barcelona Liver Cancer staging; BMI, body mass index; HR, hazard ratio.

### Perioperative safety assessment and adverse events

Perioperative safety was further evaluated across the different preoperative conversion therapy duration groups. A total of 257 patients were included, with 46 in the <12-week group, 148 in the 12–24-week group, and 63 in the >24-week group. Overall, the incidence of severe postoperative surgical complications was low. In the entire cohort, active postoperative intra-abdominal bleeding occurred in 2 patients (0.8%), bile leakage in 7 patients (2.7%), and poor wound healing in 6 patients (2.3%). The incidences of these complications were low across the three treatment-duration groups.

Among postoperative recovery-related complications, postoperative pulmonary infection occurred in 55 patients (21.4%), postoperative ascites in 155 patients (60.3%), and postoperative pleural effusion in 159 patients (61.9%). The incidences of postoperative ascites in the <12-week, 12–24-week, and >24-week groups were 58.7%, 59.5%, and 63.5%, respectively, and the corresponding incidences of postoperative pleural effusion were 65.2%, 59.5%, and 65.1%. These distributions were similar among the three groups, and no clear increasing trend was observed with longer conversion therapy duration.

Most patients experienced low-grade treatment-related AEs. In the overall cohort, grade 1 AEs occurred in 241 patients (93.8%), grade 2 AEs in 15 patients (5.8%), and grade 4 AEs in only 1 patient (0.4%); no grade 5 AEs were observed. Grouped analysis showed that the proportions of grade 1 AEs in the <12-week, 12–24-week, and >24-week groups were 95.7%, 93.2%, and 93.7%, respectively, and the incidence of high-grade AEs was low in all groups (**Table 4**).

**Table 4:** Perioperative safety according to preoperative conversion therapy duration.

Variable	Overall (n=257)	<12 weeks (n=46)	12–24 weeks (n=148)	>24 weeks (n=63)	P value
Active postoperative intra-abdominal bleeding	2 (0.8)	1 (2.2)	1 (0.7)	0 (0.0)	0.43
Bile leakage	7 (2.7)	1 (2.2)	4 (2.7)	2 (3.2)	0.95
Poor wound healing	6 (2.3)	2 (4.3)	3 (2.0)	1 (1.6)	0.59
Postoperative pulmonary infection	55 (21.4)	12 (26.1)	31 (20.9)	12 (19.0)	0.66
Postoperative ascites	155 (60.3)	27 (58.7)	88 (59.5)	40 (63.5)	0.83
Postoperative pleural effusion	159 (61.9)	30 (65.2)	88 (59.5)	41 (65.1)	0.65
Treatment-related adverse events, grade					0.91
Grade 1	241 (93.8)	44 (95.7)	138 (93.2)	59 (93.7)	
Grade 2	15 (5.8)	2 (4.3)	9 (6.1)	4 (6.3)	
Grade 3	0 (0.0)	0 (0.0)	0 (0.0)	0 (0.0)	
Grade 4	1 (0.4)	0 (0.0)	1 (0.7)	0 (0.0)	
Grade 5	0 (0.0)	0 (0.0)	0 (0.0)	0 (0.0)	

Values are presented as n (%).

Overall, the distributions of postoperative bleeding, bile leakage, poor wound healing, pulmonary infection, ascites, pleural effusion, and AE grade were similar among the preoperative conversion therapy duration groups, with no apparent trend toward perioperative safety deterioration. These findings suggest that, among patients with initially uHCC who obtained an opportunity for surgery, appropriate extension of preoperative conversion therapy did not clearly increase the risk of postoperative complications or high-grade adverse events, and hepatectomy after conversion therapy demonstrated acceptable perioperative safety.

## Discussion

This study addressed the clinically important issue of optimal surgical timing after conversion therapy for initially unresectable hepatocellular carcinoma (uHCC) by integrating two key dimensions: major pathological response (MPR) and preoperative treatment duration. The main findings were threefold. First, restricted cubic spline (RCS) analysis identified residual viable tumor (RVT) ≤15% as a clinically meaningful threshold for defining MPR, which was associated with improved overall survival (OS) and recurrence-free survival (RFS). Second, longer preoperative conversion therapy was associated with a higher probability of achieving MPR; however, the incremental survival benefit appeared to diminish beyond 24 weeks. Third, a treatment-to-surgery interval of 12–24 weeks may represent a reasonable therapeutic window that balances pathological response, survival benefit, and perioperative safety. These findings suggest that surgical decision-making after conversion therapy should move beyond determining whether resection is technically feasible and toward identifying when resection is most appropriate.

Current assessment of successful conversion therapy largely relies on radiological response and R0 resection rates (21). However, radiological complete response (CR) does not necessarily correspond to pathological complete response (pCR), and radiological resectability may not fully reflect the depth of tumor response (22). In contrast, postoperative RVT provides a direct pathological measure of residual tumor burden and may better capture long-term oncological risk (23). In this study, RCS analysis identified a risk inflection point at approximately 15% RVT, above which the risks of death and recurrence increased substantially. Accordingly, RVT ≤15% was defined as MPR. This threshold is data-driven, clinically interpretable, and potentially useful for postoperative risk stratification.

Our findings contribute to the evolving definition of MPR in HCC. Previous neoadjuvant or conversion therapy studies have commonly defined MPR as RVT ≤10% or ≥90% tumor necrosis (14, 15). However, HCC treated with combined locoregional and systemic conversion therapy often exhibits complex pathological changes, including mixed necrosis, fibrosis, inflammatory infiltration, and scar-like remodeling (24). Therefore, MPR criteria derived from other solid tumors or single-modality treatment settings may not be directly applicable to HCC after multimodal conversion therapy. The RVT ≤15% threshold identified in this study may better reflect the pathological characteristics of HCC after combined locoregional therapy, tyrosine kinase inhibitors, and immune checkpoint inhibitors. These results support the need for treatment-specific and disease-specific MPR definitions in HCC.

Importantly, this study positions MPR not only as a postoperative prognostic marker but also as a key intermediate variable linking preoperative treatment duration to surgical timing. Traditionally, the goal of conversion therapy has been to render initially unresectable tumors technically resectable (25). Our results suggest that this goal should be expanded to include the achievement of sufficient pathological response before surgery. In this context, MPR may serve as a quantifiable pathological target for optimizing the timing of resection.

This study demonstrated a non-linear relationship among treatment duration, MPR, and survival outcomes. Although prolonging conversion therapy increased the likelihood of achieving MPR, this increase did not translate into a proportional survival benefit.

Among patients treated for less than 12 weeks, the MPR rate was only 41.3%, suggesting that premature surgery may leave a substantial residual viable tumor burden. Insufficient pathological response may partly explain the poorer OS observed in this group, potentially through persistent micrometastatic disease, residual resistant clones, or inadequate systemic tumor control (26). These findings indicate that surgery performed too early after conversion therapy may compromise long-term oncological outcomes, even when technical resectability has been achieved.

In contrast, the MPR rate increased markedly to 66.2% among patients treated for 12–24 weeks, and this interval was associated with the most favorable OS. These results suggest that 12–24 weeks may represent a period during which the benefits of continued tumor response are most likely to outweigh the risks of treatment delay. However, among patients treated for more than 24 weeks, the MPR rate increased only modestly to 73.0%, and no additional RFS benefit was observed. This finding suggests a plateau effect; whereby further prolongation of treatment may deepen pathological response in some patients but may not yield additional survival benefit.

Several mechanisms may explain this phenomenon. First, treatment-sensitive tumor cells may have been largely eliminated during the earlier phase of therapy, whereas residual viable cells after prolonged treatment may represent intrinsically resistant clones (27). Second, cumulative hepatotoxicity, immune-related adverse events, aggravated fibrosis, or declining liver functional reserve may offset the potential benefit of further tumor regression (28). Third, prolonged waiting may increase the risk of disease progression or loss of the optimal surgical window (29). Therefore, the assumption that longer conversion therapy necessarily leads to better outcomes should be reconsidered. Instead, our data support the concept of an optimal therapeutic window, in which pathological response is sufficiently deepened while the risks associated with treatment delay remain acceptable.

These findings provide a practical framework for multidisciplinary decision-making after conversion therapy. Once technical resectability has been achieved, the decision to proceed with surgery should not be based solely on anatomical feasibility. Rather, the multidisciplinary team should also consider whether the patient is likely to achieve or approach MPR with additional therapy and whether continued treatment remains safe.

For patients with good treatment tolerance, preserved liver function, and ongoing tumor response but a relatively short treatment duration, such as less than 12 weeks, extending conversion therapy may be reasonable to increase the probability of achieving a deeper pathological response. Conversely, for patients who have already received 12–24 weeks of treatment and demonstrate stable or favorable radiological and serological responses, timely surgery should be actively considered to avoid unnecessary treatment delay. For patients treated beyond 24 weeks, continued therapy should be individualized and carefully weighed against potential hepatotoxicity, treatment-related adverse events, operative difficulty, and the risk of losing the surgical window.

The 12–24-week interval should therefore be regarded as a reference window rather than a rigid cutoff. Individualized surgical timing should integrate response kinetics, including AFP or PIVKA-II decline and imaging evidence of tumor necrosis or shrinkage; safety indicators, including liver function, treatment-related toxicity, and performance status; and tumor biological features, including vascular invasion, suspected microvascular invasion, and aggressive disease behavior (30). This approach may help avoid both premature resection with insufficient pathological response and ineffective prolongation of therapy without additional survival benefit.

MPR may also have important implications for postoperative management. Patients who fail to achieve MPR, particularly those with a high residual viable tumor (RVT) burden, may represent a population at high risk of recurrence and therefore require closer surveillance and consideration of postoperative adjuvant therapy. Conversely, patients who achieve MPR generally have a more favorable prognosis, and treatment de-escalation strategies could be explored in future studies. The value of adjuvant therapy after conversion hepatectomy remains controversial. Some high-quality clinical studies have shown that postoperative adjuvant therapy does not significantly improve recurrence-free survival (RFS); some have even reported numerically worse RFS in the adjuvant therapy group, although the differences did not reach statistical significance (31, 32). In contrast, other studies have suggested that postoperative adjuvant therapy may improve RFS (33, 34). Notably, our study also found an association between postoperative adjuvant therapy and worse RFS; however, this finding should be interpreted with caution, as it is more likely attributable to confounding by indication—whereby patients at higher risk of recurrence were more likely to receive adjuvant therapy—rather than a detrimental effect of adjuvant therapy itself. Therefore, decisions regarding postoperative adjuvant therapy should be based on a refined assessment incorporating the degree of pathological response, RVT burden, vascular invasion, and other recurrence-related risk factors to identify patients who are most likely to benefit.

This remains an important unresolved issue in global clinical research on HCC.This study has several limitations. First, only patients who achieved successful conversion and underwent surgical resection were included, whereas those who failed conversion therapy, experienced disease progression during treatment, or lost the opportunity for surgery were excluded, potentially introducing selection bias. Therefore, our findings apply specifically to patients with HCC who achieved successful conversion and underwent resection and should not be generalized to all patients receiving conversion therapy. Second, the targeted agents and immune checkpoint inhibitors used in this study were heterogeneous. Differences in antitumor activity among treatment combinations may have affected the observed MPR rate, and residual confounding cannot be fully excluded because treatment selection was influenced by liver function, tumor characteristics, drug availability, financial circumstances, patient preferences, and physician experience. Third, the absence of a significant difference in RFS among the treatment-duration groups may be attributable to the limited sample size, insufficient follow-up, variations in postoperative management, and imbalances in adjuvant therapy. Fourth, TARE was not routinely available during the study period because of economic, resource, and technical constraints; therefore, the applicability of our findings to TARE-treated populations remains uncertain. Finally, the RVT ≤15% cutoff and the proposed 12–24-week surgical window require external validation in larger prospective cohorts across different treatment modalities.

Future studies should prospectively include both successfully and unsuccessfully converted patients, adopt standardized treatment regimens, and validate surgical decision-making strategies based on MPR and predefined treatment windows. Predictive models integrating radiomics, circulating tumor DNA, dynamic serum biomarkers, and imaging response may help estimate the probability of achieving MPR before surgery.

## Conclusions

In summary, RVT ≤15% may serve as a prognostically meaningful MPR criterion after conversion therapy for initially uHCC, and MPR was independently associated with improved OS and RFS. Although extending preoperative conversion therapy increased the likelihood of achieving MPR, prolonging treatment beyond 24 weeks did not confer an additional RFS benefit. Based on these findings, the 12–24-week interval may represent a relatively optimal surgical window that balances pathological response, survival benefit, and perioperative safety.

## Data Availability

The raw data supporting the conclusions of this article will be made available by the authors, without undue reservation.
